# Investigating climatic changes of the wind regime over Western Iran

**DOI:** 10.1186/s13104-020-05275-z

**Published:** 2020-09-15

**Authors:** Zohreh Maryanaji, Omid Hamidi

**Affiliations:** 1 Department of Geography, Sayyed Jamaleddin Asadabadi University, 6541835583 Asadabad, Iran; 2 Department of Science, Hamedan University of Technology, 65155 Hamedan, Iran

**Keywords:** Wind regime variability, Harmonics, west of iran, Local winds, Homogenization, Spectral analysis, Daily cycle

## Abstract

**Objective:**

The aim of the present study was to reveal changes in the wind regime by investigating wind-speed data from meteorological stations in western Iran and comparing them in the last three decades (1986–2015).

**Results:**

Two main groups of daily cycles were identified; one group with a single peak and one group with two or more peaks. Using spectral decomposition technique, it was revealed that the heterogeneity observed in the area in terms of altitude and topography results in differences in the density of the spectra with similar frequencies. Two main daily cycles were also identified for each station. Although there were low frequencies, the intensity of the waves at the examined stations was the consequence of the interaction between the frequency, period, and distribution space. By evaluating harmonics in the area, it was revealed that the variance of the first harmonic is maximized in the south and southwest, while the variance of the second harmonic is maximized in the north and northwest. The positive value ​​of the trend in the first harmonic indicated that the trend of the variance for the first harmonic has increased in the central and eastern parts and has decreased in the northern and western parts.

## Introduction

The increasing urbanization rate, along with the expansion of consumerism and industrial development, have led to an increase in energy consumption in human societies. Fossil fuels are currently the main source of energy worldwide [1]. This leads to the instability of environmental conditions and reduces the success of long-term plans because of the non-renewable nature of these fuels [2].

Climate change is one of the key components of sustainable development, and human needs to recognize these changes to deal with natural destructive phenomena to harness these natural powers including wind for the benefit of mankind [3]. Understanding changes in the wind regime helps human to make use of natural environment and to restrain the wind energy, effectively. Wind plays a very important role in agriculture, urbanization, environment, transportation, energy supply, etc. [4]. Therefore, as one of the climatic elements, identification of temporal–spatial patterns of wind variability is of greate importance [4, 5]. Several studies have been conducted about wind-speed and direction [6–10].

Iran is a vast country with a major part in a warm-dry climate that is heavily influenced by climate change. In Western Iran, with over five milion residents, the development and prosperity of economy depend on the energy supply, especially renewable energies. Considering the dense population of the region and, the capacity of the energy and power production through wind forces, determination of climatic change of the wind-speed and its characteristics makes it easier to develop the region economically/socially. Therefore, displaying the hidden features of the wind through harmonic analysis has many applications in management and planning of energy in the region. In this study, the variability of the wind regime was investigated over the past three decades in western Iran using different harmonics and Fourier analysis. Moreover, the daily cycles of the wind-speed variations were detected using spectral analysis.

## Main text

### Materials and methods

#### Geographical location of the study area

**Fig. 1:**
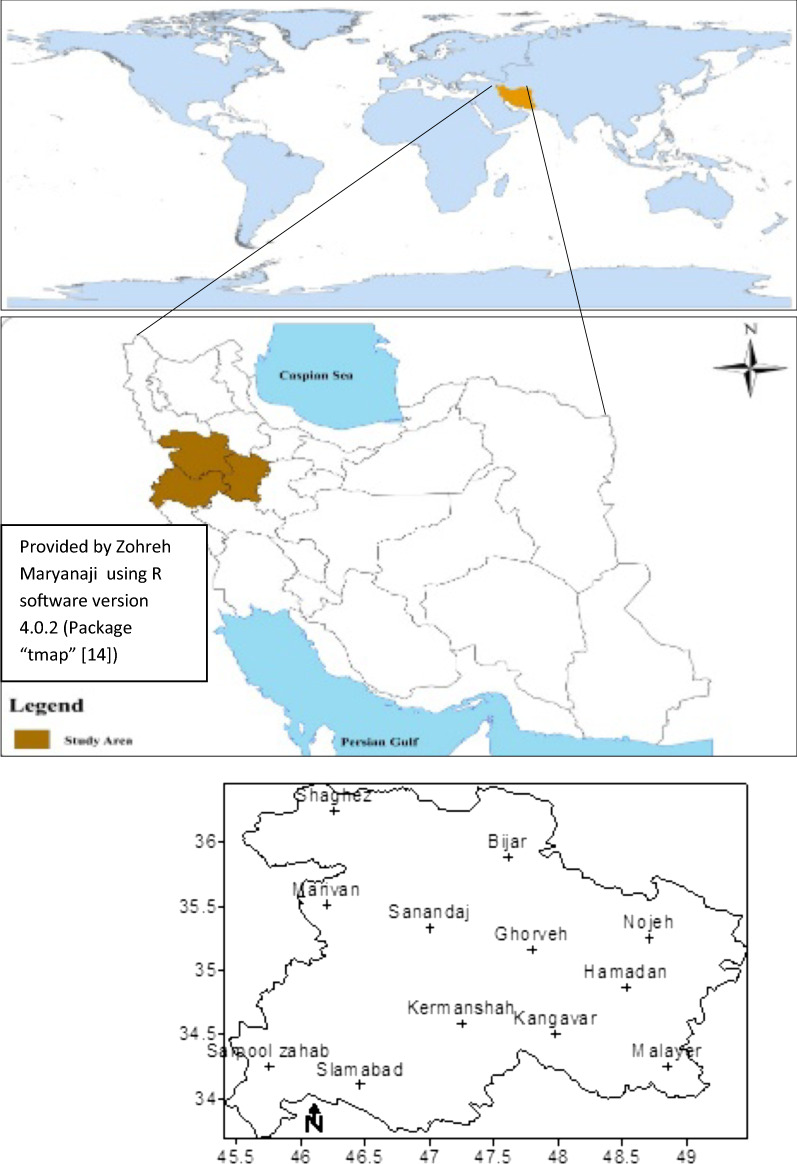
Location of the studied area; Provided by Zohreh Maryanaji using R software version 4.0.2 (Package “tmap” [14], open source software)

The study area is located in a mountainous region of western Iran (the northern parts of Zagros altitudes, 73,503 km^2^), between the 33° to 37° north latitude and 45° to 50° east longitude of Greenwich meridian (Fig. 1) [11]. In this study, the hourly wind-speed data (meter/second) over a thirty-year peiod (1986–2015) were used from synoptic stations (observational). (Additional file 1. Table S1) shows the characteristics of the stations.

#### Data analysis

Preprocessing and quality control of the wind-speed time-series data were provided in Additional file 2. shows a heterogenous and homogenized time series in a sample station. The correction coefficients used for homogenization wind-speed data were provided for all heterogenous stations (Additional file 1. Table S2). The “WINDOGRAPHER” software [12] was used to investigate the daily wind-speed variability, illusterate the wind-speed graphically in different months and display the peak of the wind-speed in the days/nights. Moreover, to show the frequencies in the daily wind cycles, the spectral analysis was utilized (using XLSTAT software [13]). Fourier analysis (harmonics) was used to study the variation of wind-regime and the role of the roughness in the wind-speed of the region. “tma” package (of R.4.0.2 software) [14].

## Results

**Fig. 2:**
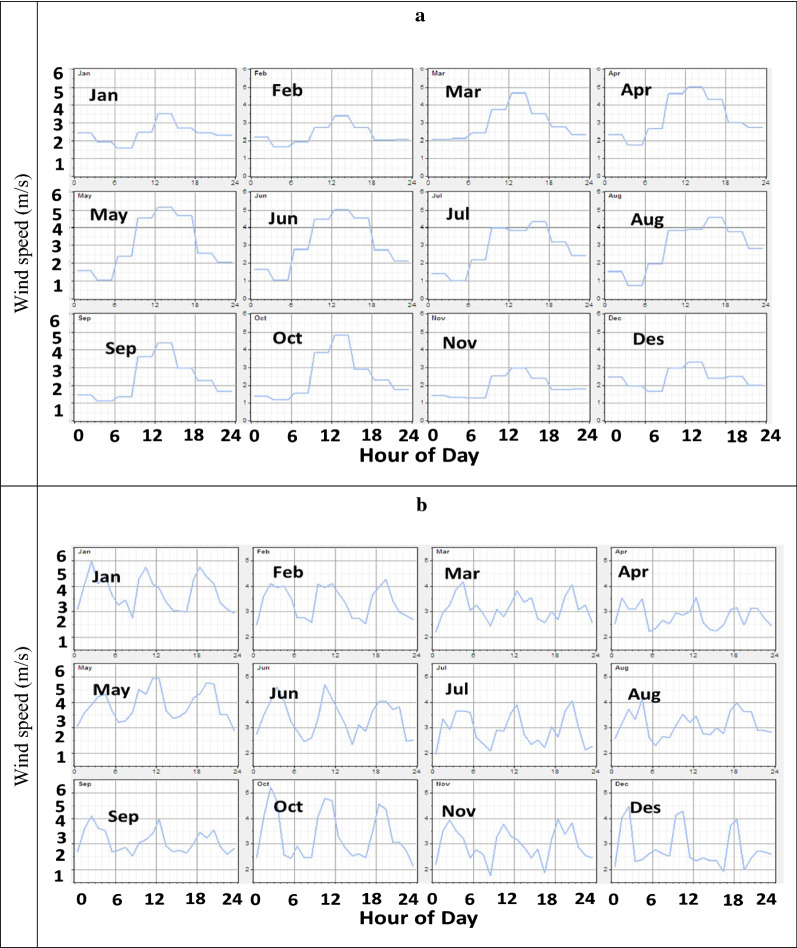
Daily cycle graphs for (**a**) Hamadan Station as a sample station of the first group and (**b**) for Malayer Station as a sample station of the second group

By investigating the daily wind-speed cycles, two main groups of stations were identified. The first group included stations with a 24-h peak (happens between 11:00–16:00) (Hamadan/Kermanshah/Marivan/Sarpool.Zahab/Nojeh/Shghez/Slamabad). Fig. 2a shows the daily cycle diagrams of the Hamadan station as a sample station. The second group includes stations with two/more peaks of wind-speed (Sanandaj/Malayer/Ghorveh/Bijar/Kangavar). Fig. 2b shows the daily cycle graphs of the Malayer station as a sample station.

**Fig. 3:**
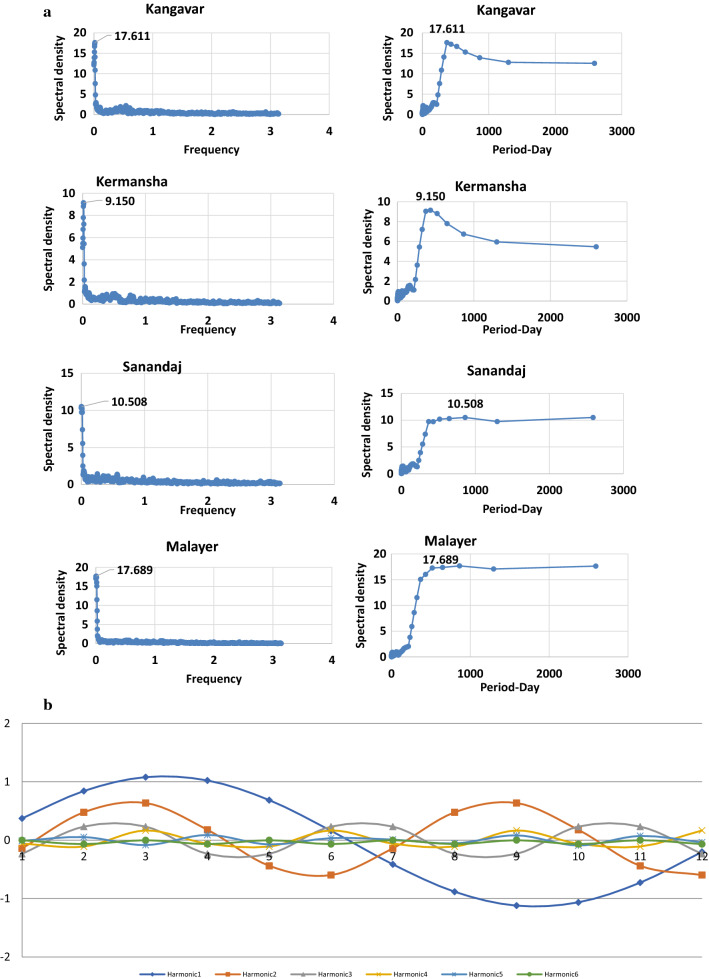
**a** Spectral density and cycles for the sample stations; and **b** Amplitude of harmonics in Hamadan sample station over the study period

The results of the spectral analysis of the whole stations were shown in Additional file 1. Table S3. As shown, the obtained primary frequencies were approximately the same for most of the stations, but the spectral densities vary across them and two main daily cycles were identified for each station. For example, in the Sanandaj station, they occurred at 0.007 and 0.017 frequencies, with a period of 863 and 463 days, respectively (with a spectral density of 10.5 and 9.74 respectively). Fig. 3a shows the spectral density and cycles for the sample stations (Sanandaj/Malayer/Kermanshah/Kangavar). As seen, for example for Sanandaj station, the highest density is related to the two mentioned periods.

Fig. 3b shows the harmonics for the Hamadan sample station obtained from *Fourier analysis*. The phase-angle indicates the time of the maximum/peak of a harmonic. The zero value indicates December 15.

As seen (Fig. 3b and Additional file 1. Table_S4), the first and the second harmonics in the area are adequate to describe the data variations (the cumulative percentage of variances ≥ 95%). Analysis of the first harmonic represents a one-year cycle which is a wave with the largest amplitude covering the whole period. The second harmonic, which consists of two waves, represents the spatio-temporal variations during the semi-annual periods which includes increasing frequency.

(Additional file 3. Figures S1–S5) depict the variance, amplitude and time of the first and secon harmonics over the three decades and the whole study-period, separately. Comparing the maps indicates that the variance of the first harmonic was greater in the south/western regions, while the variance of the second harmonic was greater in the north/northwest of the study area. According to the maps, the variance of the first harmonic is over 60% throughout the studied area. This quantity becomes 85% in the southwest parts of the area and its magnitude increases from Northwest towards South/Southwest. The magnitude of the variance of the second harmonic is greater (over 26%) in the north/northwest and it is reduced towards South.

Additional file 3. Figure S6 shows the trends of the magnitudes of two harmonics which are positive (negative) over the central/eastern (north/northwest) parts of the area for the first (second) harmonic and negative (positive) in other regions. It was also observed that the trend of time values in the first harmonic was slightly decreasing in the western parts of the area. The magnitude of the amplitudes was not significantly different in three decades.

## Discussion and conclusion

This study showed that despite the existence of the same meteorological systems throughout the studied area, the main factors that cause differences in the spectral densities in the stations are the topographic conditions and roughness of the earth [15]. By investigating the periodic behavior of the wind regime, it was revealed that the first harmonic is an indication of winds related to pressure variations in masses and air systems on a synoptic scale. These masses, formed beyond the geographical boundaries, enter the area in the winter with the arrival of the westerlies. This is the reason that the severe winds, clouds growth and precipitation are usually accompanied by low pressure masses. In summers, due to the direct sunlight on the desert areas of the central Iran and the warming up of the adjacent air in the surface of the earth, the low-pressure system is dominant in these areas and because of the instability resulting from the establishment of this low-pressure system, usually strong winds and dust are prevailed. In the southern/central parts of the area, the first harmonic is more effective than the other parts. Its effect is larger than the overseas systems and is lower than roughness. Although, in some eastern/northwest parts of the studied area (Alvand Mountains), the magnitude of the second harmonic was greater compared with the other parts (which expresses the impact of topography and local conditions on wind), the magnitude of the variance of the second harmonic in these areas was ≥ 25%. Local winds occurrence are affected by local phenomena like the mountainous/topographic effects of the region. As the study area is located in the Zagros mountain range, which has high summits, it is exposed to the plain-mountain winds. The proximity of the plains to the highlands/mountainous areas increases the second harmonic of the wind in this region and creates local winds. By comparing harmonic changes in the first, second and third decades, it seems that the second harmonic has an increasing contribution in the region, which means that the wind regime in these areas is mostly affected by local factors and features of the earth’s surface and the effects of the systemic winds (synoptic pressure difference) have been reduced by an average of 3–7% in the areas.

Trend analysis of the harmonics indicated that topography and altitude are important factors in establishment of wind and the maximum wind-speed happens in east/northeast regions (largerest amplitudes). Our findings, also, showed that the time of the maximum wind-speed starts (finishes) from early (late) autumn. Deacreasing (increasing) the variance of the first (second) harmonic in the second decade compared with the first decade indicates an increase in local winds and in the contribution of geographic factors in wind formation. Moreover, in the second decade, the time of the maximum wind-speed for large-scale systems occurs 15 days later than the first decade. This can be attributed to the climatic/atmospheric changes in a macro scale. The time of the occurrence of the maximum wind-speed in the second harmonic is in late November which is due to the cooling of the altitudes and establishing thermal high-pressures at altitudes. The magnitude of the amplitudes of the second harmonic was greater in the East/Northeast, i.e. the maximum wind-speed happens in these areas and it tends to decrease from the northeast towards the southwest.

Assessing the periodic behavior of the wind indicated the type of the wind regime and its influencibility by the altitudes. Comparing the results of the harmonic analysis over a thirty-year period indicates that in the study area, altitudes play a major role in establishment of wind blowing and its maximum wind-speed especially in the northern and eastern parts of the area.

By evaluating the trends for the magnitude of the amplitudes (maximum daily wind-speed), it was found that the trend is slightly decreasing in the first harmonic and it is increasing with the same amount in the second harmonic. This can be interpreted as follows: as the systemic winds blows, that is a function of large-scale systems (synoptic), topography and high mountains play a significant role in accelerating the wind-speed.

By investigating wind regime changes using harmonic analysis in the present study, it was indicated that by increasing the contribution of the variance of the second harmonic, the altitudes and geographic factors contribute more to the wind establishment. For winds with a systemic origin and large-scale systems, the occurrence time of the maximum wind-speed has changed from early November to mid-November. This delay is due to the delays in the entrance of westerlies and extratropical systems to the region, and it might be related to the climate change and global warming. Nevertheless time has not changed in the second harmonic. The results of this research can be applied in the management of energy resources and environmental planning. It is suggested to investigate the changes and displacement in the wind regime in different regions of Iran.

Since the values of the first harmonic are affected due to the arrival of exterior (synoptic) systems in the region and it is variable (although its values are larger than the second harmonic), planning based on this harmonic is associated with high risk. On the contrary, because the values of the second harmonic are affected by the ripples and the topography of the region and this harmonic is a constant geographic factor, therefore, planning based on this harmonic is associated with lower risk. Accordingly, in areas where the second harmonic values are higher in the wind regime, the development and installation of wind turbines and long-term planning for the use of wind energy are more logical. Thus, it can be concluded that the eastern and northwest regions of the study area are more suitable for the use of wind energy as well as the development and installation of wind turbines and it can play a significant role in the economic and social prosperity of the region.

## Limitations

Lack of long-term data registery.

## Supplementary information

**Additional file 1:** **Table S1.** Characteristics of the meteorological stations of the study area. **Table S2.** Correction coefficients used for homogenization wind-speed data. **Table S3.** The results of spectral analysis for the wind-speed data for studied stations. **Table S4.** Variance, amplitude and the time of the occurrence of the maximum wend-speed in the first and second harmonics.

**Additional file 2.** Assessment of the data homogeneity and quality control of data.

**Additional file 3:** **Figure S1.** Homogenization of the wind-speed data at Nojeh station (sample station): the top chart shows the heterogeneous time series of wind-speed; the middle chart shows the trend of heterogeneous time series of wind-speed indicating a jump in 1989 due to the displacement of the station; and the bottom chart shows the homogenized wind-speed. **Figure S2.** Variance (V), amplitude (A) and time (T) of the harmonics (numbers 1 and 2 indicates first and second harmonics) for the average wind-speed in the study area over a 30 year period (1986–2015) (phase angle or T the time in the bottom panels indicate the change in the time of maximum (peak) of the harmonics; A zero value indicates (15th December, − 0.5 stands for 15 days earlier, i.e. December 1 and 0.5 shows 15 days later, i.e., December 30). **Figure S3.** Variance (V), amplitude (A) and time (T) of the harmonics (numbers 1 and 2 indicates first and second harmonics) of the average wind-speed in the study area over the first decade (1986–1995) (phase angle or T the time in the bottom panels indicate the change in the time of maximum (peak) of the harmonics; A zero value indicates (15th December, − 0.5 stands for 15 days earlier, i.e. December 1 and 0.5 shows 15 days later, i.e., December 30). **Figure S4.** Variance (V), amplitude (A) and time (T) of the harmonics (numbers 1 and 2 indicates first and second harmonics) of the average wind-speed in the study area over the second decade (1996–2005) (phase angle or T the time in the bottom panels indicate the change in the time of maximum (peak) of the harmonics; A zero value indicates (15th December, − 0.5 stands for 15 days earlier, i.e. December 1 and 0.5 shows 15 days later, i.e., December 30). **Figure S5.** Variance (V), amplitude (A) and time (T) of the harmonics (numbers 1 and 2 indicates first and second harmonics) of the average wind-speed in the study area over the third decade (2006–2015) (phase angle or T the time in the bottom panels indicate the change in the time of maximum (peak) of the harmonics; A zero value indicates (15th December, − 0.5 stands for 15 days earlier, i.e. December 1 and 0.5 shows 15 days later, i.e., December 30). **Figure S6.** The magnitude of the trends for the Variance (V), amplitude (A) and time (T) of the harmonics (numbers 1 and 2 indicates first and second harmonics) of the average wind-speed in the study area over the thirty-year period (1986–2015) (phase angle or T the time in the bottom panels indicate the change in the time of maximum (peak) of the harmonics; A zero value indicates (15th December, − 0.5 stands for 15 days earlier, i.e. December 1 and 0.5 shows 15 days later, i.e., December 30).

## Data Availability

The data is available upon the request from the first author.
