# Supplementary material for: Investigating climatic changes of the wind regime over Western Iran
Source: BMC Res Notes. 2020 Sep 15;13:434. doi: 10.1186/s13104-020-05275-z (PMC7493849; doi:10.1186/s13104-020-05275-z)
Supplement: Supplementary file 1 — Additional file 1: Table S1. Characteristics of the meteorological stations of the study area. Table S2. Correction coefficients used for homogenization wind-speed data. Table S3. The results of spectral analysis for the wind-speed data for studied stations. Table S4. Variance, amplitude and the time of the occurrence of the maximum wend-speed in the first and second harmonics. [file 13104_2020_5275_MOESM1_ESM.docx]

Additional Tables

Table S1. Characteristics of the meteorological stations of the study area

| Station | Altitude (m) | Longitude | latitude |
| --- | --- | --- | --- |
| Slamabad | 1348 | 46° 28' | 34° 07' |
| Kermanshah | 1318 | 15° 47 ' | 34° 35 ' |
| Sarpool Zahab | 545 | 45° 52 ' | 34° 27 ' |
| Hamadan Nojeh | 1679 | 43° 48 ' | 15° 35 ' |
| Hamadan Airport | 1741 | 48° 32 ' | 34° 52 ' |
| Malayer | 1777 | 48° 51 ' | 34° 15 ' |
| Kangavar | 1468 | 47° 59 ' | 34° 30 ' |
| Sanandaj | 1373 | 47° 00 ' | 35° 20 ' |
| Sheghez | 1522 | 46° 16 ' | 36° 15 ' |
| Marivan | 1286 | 46° 12 ' | 35° 31 ' |
| Ghorveh | 1906 | 47° 48 ' | 35° 10 ' |
| Bijar | 1883 | 47° 37 ' | 35° 53 ' |

Table S2. Correction coefficients used for homogenization wind-speed data

| Station name | Correction Coefficient |
| --- | --- |
| Sarpool Zahab | 0.51 |
| SlamAbad | 0.75 |
| Nojeh | 0.74 |
| Malayer | 0.70 |
| Marivan | 0.80 |
| Bijar | 0.58 |

Table S3. The results of spectral analysis for the wind-speed data for studied stations

|  | 24 hours Peaks | | | 12 hours peak | | |
| --- | --- | --- | --- | --- | --- | --- |
| Station name | Frequency | Period  (in day) | Spectral density | Frequency | Period  (in day) | Spectral density |
| Hamadan | 0.017 | 364 | 4.306 | 0.035 | 182 | 3.057 |
| Nojeh | 0.015 | 432 | 9.150 | 0.017 | 371 | 9.058 |
| Kermanshah | 0.015 | 432 | 9.150 | 0.017 | 371 | 9.058 |
| Kangavar | 0.017 | 370 | 17.611 | 0.015 | 432 | 17.210 |
| Ghorveh | 0.017 | 370 | 28.640 | 0.005 | 1295 | 37.110 |
| Marivan | 0.007 | 863 | 17.680 | 0.005 | 1295 | 17.630 |
| Malayer | 0.007 | 863 | 17.689 | 0.017 | 370 | 15.070 |
| Sanandaj | 0.007 | 863 | 10.500 | 0.017 | 463 | 9.740 |
| Slamabad | 0.012 | 420 | 17.600 | 0.015 | 332 | 15.210 |
| Sarpool Zahab | 0.015 | 490 | 18.500 | 0.020 | 360 | 9.690 |
| Sheghez | 0.005 | 895 | 27.310 | 0.025 | 263 | 12.600 |
| Bijar | 0.015 | 785 | 14.300 | 0.019 | 363 | 11.900 |

Table S4. Variance, amplitude and the time of the occurrence of the maximum wend-speed in the first and second harmonics

|  |  |  |  |  |  |  |
| --- | --- | --- | --- | --- | --- | --- |
| Station | Variance 1 | Amplitude | time | Variance2 | Amplitude | time |
| Hamadan | 68.9 | 1.2 | -2.6 | 22.75 | 0.7 | -0.26 |
| Malayer | 70.9 | 0.75 | -2.6 | 20.5 | 0.4 | -0.7 |
| Nojeh | 77.3 | 1.3 | -1.8 | 12.38 | 0.5 | -1 |
| Kangavar | 73.58 | 0.95 | -1.5 | 17.77 | 0.5 | -0.6 |
| Slamabad | 87.97 | 1 | -0.78 | 8.11 | 0.3 | -1.3 |
| Kermanshah | 79.6 | 0.92 | -2 | 15.5 | 0.4 | -1 |
| Sarpool zahab | 85.7 | 0.8 | -1 | 12 | 0.3 | 0.3 |
| Sanandaj | 81.4 | 0.9 | -1.8 | 14.7 | 0.4 | -0.7 |
| Shaghez | 60.74 | 0.9 | -1.7 | 36.32 | 0.7 | -0.5 |
| Marivan | 61.8 | 0.7 | -1.4 | 35 | 0.54 | -0.6 |
| Ghorveh | 71.2 | 1.3 | -2.5 | 26.3 | 0.8 | -0.3 |
| Bijar | 67.5 | 1.6 | -2.9 | 30.2 | 1 | -0.1 |

*zero values indicate the December 15, negative (positive) values indicate dates before (after) the December 15
