# Supplementary material for: Investigating climatic changes of the wind regime over Western Iran
Source: BMC Res Notes. 2020 Sep 15;13:434. doi: 10.1186/s13104-020-05275-z (PMC7493849; doi:10.1186/s13104-020-05275-z)
Supplement: Supplementary file 2 — Additional file 2. Assessment of the data homogeneity and quality control of data. [file 13104_2020_5275_MOESM2_ESM.docx]

*Assessment of the data homogeneity and quality control of data*:

Before doing any calculations, the homogeneity of the data was tested and the accuracy of the data was assured. In this regard, before performing any calculation, homogeneity test was performed to verify the accuracy and homogeneity of the data. Therefore, the Two‐Phase Regression (TPR) test and its modified algorithm called PMFRED test (a common trend two-phase regression-model-based test; the extended version of the penalized maximal F test, or ‘‘PMFred,’’ algorithm) were used to assess the homogeneity of the wind-speed time series data [[1](#_ENREF_1)]. The homogeneity test was performed at a confidence level of 99% and change points or jumps in the studied stations were assessed. The method is as follows:

To test the potential jumps or heterogeneity in the time series at time *k* with linear trend *β*, the following null and alternative hypothesis were considered:

$\begin{matrix} H_{0}:X_{t}=\mu+\beta t+\varepsilon_{t}\begin{matrix} & t=1,2,\ldots,N & \end{matrix} \\ H_{a}:\left\{\begin{matrix} X_{t}=\mu_{1}+\beta t+\varepsilon_{t} & t\leq k \\ X_{t}=\mu_{2}+\beta t+\varepsilon_{t} & k-1\leq t\leq N \end{matrix}\right.. \end{matrix}$ (S1)

If the null hypothesis is correct, the most probable point for occurrence of the jump is $F_{\max}=\max_{1\leq k\leq N-1} \frac{\left(SSE_{0}-SSE_{A}\right)}{SSE_{A}/(N-3)}$ (S2)

where

$SSE_{A}=\sum_{t=1}^{k} \left(X_{t}-\hat{\mu}_{1}-\hat{\beta}t\right)^{2}+\sum_{t=k+1}^{N} \left(X_{t}-\hat{\mu}_{2}-\hat{\beta}t\right)^{2}$ (S3)

and

$SSE_{0}=\sum_{t=1}^{N} \left(X_{t}-\hat{\mu}_{0}-\hat{\beta}t\right)^{2}$. (S4)

where SSEs stand for sum of square errors. If there is no jump in the data, the errors will have normal distribution and *F_max_* will have F distribution. If *F_max_* for a time series shows the maximum critical value at the predefined confidence level, there will be a jump or a change point in the data, otherwise the series will be homogeneous [[2](#_ENREF_2)].

In the case there is a jump, the P(K) method was used to adjust the data using the following equation:

$PF_{\max}=\max_{1\leq k\leq N-1}\left[P(k)F_{c}(k)\right]$ (S5)

Moreover, the PMFRED algorithm was used to incorporate the autocorrelations in the data [[3](#_ENREF_3)].

The heterogeneity in the data was adjusted using a correction coefficient calculated by dividing the average wind-speed at the time period after the change point to the average of the wind-speed at the time period before the change point [[4](#_ENREF_4)]. This coefficient was applied for all data before the change point.

The data obtained from Nojeh, Slamabad, Malayer, Sarpool Zahab, Bijar and Marivan stations were diagnosed as heterogeneous. There are several potential sources of heterogeneity for the wind data in Iran, including changing the measurement methods during the period of recording wind data, changing the environment conditions around the meteorological stations, the displacement of the station locations (especially from urban areas to the countryside), the changes in the height and construction of the buildings near the stations, the change in the surrounding vegetation. The main synoptic stations, such as Hamadan and Kermanshah, are among these stations. As a result, it should be expected that the heterogeneity observed in the data set is due to the displacement of the station locations from urban areas to the countryside. Moreover, the discontinuity in long-term data and insufficient information about the history of stations are among the main problems of homogeneity of wind data in Iran. So, the correction coefficients (shown in Table S2) were applied to them to alleviate this heterogeneity. To depict the homogenization process of the data, the Nojeh station was selected as a sample station (Figure S1). As seen, there was a jump in the data at 1989 and using the correction coefficient of 0.74, the data was homogenized.

Moreover, for statistical indices assessment some primary indicators such as mean, variance, coefficient of variation, and standard deviation were calculated. For the outliers, we used trimmed mean.

*Spectral analysis*

To investigate the daily wind-speed variability and to illustrate the wind-speed graphically in different months as well as to display the peak of the wind-speed in the days and nights, the “WINDOGRAPHER” software was used. Moreover, to show the frequencies in the daily wind cycles the spectral analysis was utilized (using XLSTAT software). In this method, the time series was first converted to frequencies, then the variance of the frequencies was calculated, and finally the periodographer or spectrum was plotted. The spectral analysis is a technique that converts the time series to frequencies and shows the cycle with the most important role. Then, after evaluation of the periods, the waves are separated according to their importance. The following equations were used to convert the fluctuations to the frequencies:

$a_{q}=\frac{2}{n}\sum_{t=1}^{n} x_{t}\cos(\frac{2\pi q}{n}t)$ (S6)

and

$b_{q}=\frac{2}{n}\sum_{t=1}^{n} x_{t}\sin(\frac{2\pi q}{n}\mathrm{t})$ (S7)

where *n* is the length of the time series, $t=1,...,n$ shows time,$q=1,...,\frac{n}{2}$ $a_{q}$and$b_{q}$ are the coefficients of the “Fourier representation” for a time series.

*Harmonic analysis*

Fourier analysis (harmonics) was used to study the variation of wind regime and the role of the roughness in the wind-speed of the region. For this purpose, using wind-speed data (meter/second) over a period of thirty years and Fourier technique, the harmonics of the wind-speed, its periodic behaviors and the temporal changes were investigated.

Any form of periodic behaviors is estimated and predicted using sinusoidal functions at different frequencies with an appropriate and acceptable approximation. In this regard, Fourier models are considered as useful and efficient tools. In fact, sinusoidal and cosine components are harmonics that are effective in the formation of periodic series behavior. The number of these harmonics (oscillating components) is, at most, half the length of the data. Because oscillatory behavior consists of at least two components (sinus and cosine), each of them indicates an upward and a downward trend in a time series. Therefore, each successive wavelength in the periodic time series is illustrated by a harmonic. Harmonics can be constructed using a time series as follows [[5](#_ENREF_5), [6](#_ENREF_6)]:

| $X_{t}=\overline{X}+\sum_{i=1}^{n} \left[a_{i}\cos\frac{2\pi}{T}it+b_{i}\sin\frac{2\pi}{T}it\right]$ | (S8) |
| --- | --- |

where $\overline{X}$ is arithmetic mean and *a_i_* and *b_i_* are the coefficients of the *i*^th^ harmonic.

It should be noted that Fourier illustration (of a finite sequence) is a purely mathematical concept, nevertheless it can be determined by a number of harmonics that are statistically significant and gets good approximations. Usually, a small number of harmonics are sufficient to explain behavior of a climatic element. As can be seen from equation (1), sinus and cosine components determine series behavior around a constant mean.

As it is clear, the Fourier pattern can also be considered as a linear regression model in which the number of regression coefficients is equal to the length of the series .In the equation S8, the coefficients are calculated using least square method. These coefficients are as follows:

| $a_{i}=\frac{2}{n}\sum_{t=1}^{n-1} x_{t}\cos\left(\frac{2\pi}{12}it\right),i=1,2,....\frac{n}{2}$ | (S9) |
| --- | --- |
| $b_{i}=\frac{2}{n}\sum_{t=0}^{n-1} x_{t}\sin\left(\frac{2\pi}{12}it\right),i=1,2,....\frac{n}{2}$ | (S10) |

where *x_t_* is the average monthly wind-speed and *n* is the number of observation. The amplitude (A), the phase angle of the i^th^ harmoni (), the time of occurrence of the maximum wind-speed fall in each harmonic follows (*t*) and the variance of each harmonic (V) can be determined as follows ((S11)-(S14)):

| $A_{i}=\sqrt{a_{i}^{2}+b_{i}^{2}}$ | (S11) |
| --- | --- |
|  | (S12) |
| $t_{i}=\left(\frac{T}{2\pi i}\right)\varphi_{i},$ | (S13) |
| $V_{i}=\frac{A_{i}^{2}}{2}.$ | (S14) |

In general, the first harmonic indicates an individual annual cycle of the observations and the second harmonic indicates the tendency toward a half-yearly change [[5](#_ENREF_5)]. Time (t), shows the change in the location of the maximums in the time axis. Analyzing the changes in the contribution of variance of different harmonics determines the amplitude (maximum amount of wind in different regions) and the time of occurrence of the maximum wind speed in the three decades of wind-speed regime changes in western Iran over the study period. Normally, there are 6 harmonics in monthly data to use and analyze the distribution of the data. However in the present study, the variances of the first two harmonics were utilized as they covered more than 95% of the variations. Therefore, the two first harmonics were used to interpret the variability of the wind regime. The trend of the wind-speed harmonics was investigated through linear regression using least square method.

1. Wang, X.L., Q.H. Wen, and Y. Wu, *Penalized maximal t test for detecting undocumented mean change in climate data series.* Journal of Applied Meteorology and Climatology, 2007. **46**(6): p. 916-931.

2. Wang, X.L., *Penalized maximal F test for detecting undocumented mean shift without trend change.* Journal of Atmospheric and Oceanic Technology, 2008. **25**(3): p. 368-384.

3. Wang, X.L., *Accounting for autocorrelation in detecting mean shifts in climate data series using the penalized maximal t or F test.* Journal of Applied Meteorology and Climatology, 2008. **47**(9): p. 2423-2444.

4. Peterson, T.C., D.R. Easterling, T.R. Karl, P. Groisman, N. Nicholls, N. Plummer, S. Torok, I. Auer, R. Boehm, and D. Gullett, *Homogeneity adjustments of in situ atmospheric climate data: a review.* International Journal of Climatology: A Journal of the Royal Meteorological Society, 1998. **18**(13): p. 1493-1517.

5. Tarawneh, Q. and M. Kadıoğlu, *An analysis of precipitation climatology in Jordan.* Theoretical and applied climatology, 2003. **74**(1-2): p. 123-136.

6. Sabziparvar, A., S. Movahedi, H. Asakereh, Z. Maryanaji, and S. Masoodian, *Geographical factors affecting variability of precipitation regime in Iran.* Theoretical and applied climatology, 2015. **120**(1-2): p. 367-376.
