# Supplementary material for: Investigating climatic changes of the wind regime over Western Iran
Source: BMC Res Notes. 2020 Sep 15;13:434. doi: 10.1186/s13104-020-05275-z (PMC7493849; doi:10.1186/s13104-020-05275-z)
Supplement: Supplementary file 3 — Additional file 3: Figure S1. Homogenization of the wind-speed data at Nojeh station (sample station): the top chart shows the heterogeneous time series of wind-speed; the middle chart shows the trend of heterogeneous time series of wind-speed indicating a jump in 1989 due to the displacement of the station; and the bottom chart shows the homogenized wind-speed. Figure S2. Variance (V), amplitude (A) and time (T) of the harmonics (numbers 1 and 2 indicates first and second harmonics) for the average wind-speed in the study area over a 30 year period (1986–2015) (phase angle or T the time in the bottom panels indicate the change in the time of maximum (peak) of the harmonics; A zero value indicates (15th December, − 0.5 stands for 15 days earlier, i.e. December 1 and 0.5 shows 15 days later, i.e., December 30). Figure S3. Variance (V), amplitude (A) and time (T) of the harmonics (numbers 1 and 2 indicates first and second harmonics) of the average wind-speed in the study area over the first decade (1986–1995) (phase angle or T the time in the bottom panels indicate the change in the time of maximum (peak) of the harmonics; A zero value indicates (15th December, − 0.5 stands for 15 days earlier, i.e. December 1 and 0.5 shows 15 days later, i.e., December 30). Figure S4. Variance (V), amplitude (A) and time (T) of the harmonics (numbers 1 and 2 indicates first and second harmonics) of the average wind-speed in the study area over the second decade (1996–2005) (phase angle or T the time in the bottom panels indicate the change in the time of maximum (peak) of the harmonics; A zero value indicates (15th December, − 0.5 stands for 15 days earlier, i.e. December 1 and 0.5 shows 15 days later, i.e., December 30). Figure S5. Variance (V), amplitude (A) and time (T) of the harmonics (numbers 1 and 2 indicates first and second harmonics) of the average wind-speed in the study area over the third decade (2006–2015) (phase angle or T the time in the bottom panels indicate the ch [file 13104_2020_5275_MOESM3_ESM.docx]

Aditional Figures

|  |
| --- |
|  |
|  |

Figure S1. Homogenization of the wind-speed data at Nojeh station (sample station): the top chart shows the heterogeneous time series of wind-speed; the middle chart shows the trend of heterogeneous time series of wind-speed indicating a jump in 1989 due to the displacement of the station; and the bottom chart shows the homogenized wind-speed

|  |  |
| --- | --- |
|  |  |
|  |  |

Figure S2. Variance (V), amplitude (A) and time (T) of the harmonics (numbers 1 and 2 indicates first and second harmonics) for the average wind-speed in the study area over a thirty-year period (1986-2015) (phase angle or *T* the time in the bottom panels indicate the change in the time of maximum (peak) of the harmonics; A zero value indicates (15^th^ December, -0.5 stands for 15 days earlier, i.e. December 1 and 0.5 shows 15 days later, i.e., December 30). Provided by Zohreh Maryanaji using R software version 4.0.2 (Package “tmap” [14], open source software)

|  |  |
| --- | --- |
|  |  |
|  |  |

Figure S3. Variance (V), amplitude (A) and time (T) of the harmonics (numbers 1 and 2 indicates first and second harmonics) of the average wind-speed in the study area over the first decade (1986-1995) (phase angle or *T* the time in the bottom panels indicate the change in the time of maximum (peak) of the harmonics; A zero value indicates (15^th^ December, -0.5 stands for 15 days earlier, i.e. December 1 and 0.5 shows 15 days later, i.e., December 30). Provided by Zohreh Maryanaji using R software version 4.0.2 (Package “tmap” [14], open source software)

| **** | **** |
| --- | --- |
| **** | **** |
| **** | **** |

Figure S4. Variance (V), amplitude (A) and time (T) of the harmonics (numbers 1 and 2 indicates first and second harmonics) of the average wind-speed in the study area over the second decade (1996-2005) (phase angle or *T* the time in the bottom panels indicate the change in the time of maximum (peak) of the harmonics; A zero value indicates (15^th^ December, -0.5 stands for 15 days earlier, i.e. December 1 and 0.5 shows 15 days later, i.e., December 30). Provided by Zohreh Maryanaji using R software version 4.0.2 (Package “tmap” [14], open source software)

| **** | **** |
| --- | --- |
| **** | **** |
| **** | **** |

Figure S5. Variance (V), amplitude (A) and time (T) of the harmonics (numbers 1 and 2 indicates first and second harmonics) of the average wind-speed in the study area over the third decade (2006-2015) (phase angle or *T* the time in the bottom panels indicate the change in the time of maximum (peak) of the harmonics; A zero value indicates (15^th^ December, -0.5 stands for 15 days earlier, i.e. December 1 and 0.5 shows 15 days later, i.e., December 30). Provided by Zohreh Maryanaji using R software version 4.0.2 (Package “tmap” [14], open source software)

|  |  |
| --- | --- |
|  |  |
|  |  |

Figure S6. The magnitude of the trends for the Variance (V), amplitude (A) and time (T) of the harmonics (numbers 1 and 2 indicates first and second harmonics) of the average wind-speed in the study area over the thirty-year period (1986-2015) (phase angle or *T* the time in the bottom panels indicate the change in the time of maximum (peak) of the harmonics; A zero value indicates (15^th^ December, -0.5 stands for 15 days earlier, i.e. December 1 and 0.5 shows 15 days later, i.e., December 30). Provided by Zohreh Maryanaji using R software version 4.0.2 (Package “tmap” [14], open source software)
